# Colorectal Cancer in the Elderly Patient: The Role of Neo-adjuvant Therapy

**DOI:** 10.1515/med-2019-0068

**Published:** 2019-08-14

**Authors:** Concetta Anna Dodaro, Armando Calogero, Vincenzo Tammaro, Tommaso Pellegrino, Ruggero Lionetti, Silvia Campanile, Marsela Menkulazi, Massimo Ciccozzi, Anna Maria Iannicelli, Francesco Giallauria, Caterina Sagnelli

**Affiliations:** 1 Department of Advanced Biomedical Sciences, University of Naples Federico II, Naples, Italy; 2 Department of Public Health, University of Naples Federico II, Naples, Italy; 3 Medical Statistics and Molecular Epidemiology Unit, Campus Bio-Medico University, Rome, Italy; 4 Department of Translational Medical Sciences, University of Naples “Federico II”, Naples, Italy; 5 Department of Mental Health and Public Medicine, University of Campania Luigi Vanvitelli, Naples, Italy

**Keywords:** Neoadjuvant chemoradiotherapy, Colorectal tumors, Rectal cancer

## Abstract

**Background:**

Neoadjuvant chemoradiotherapy has a significant role in downstaging cancer. It improves the local control of the disease and can make conservative resection of rectal cancer possible.

**Methods:**

We enrolled 114 patients with subperitoneal rectal cancer who underwent neoadjuvant chemoradio-therapy and radical excision with total mesorectal excision (TME). The primary endpoint was oncological outcomes and the secondary endpoint was surgical outcomes.We evaluate the experience of a multidisciplinary team and the role of neoadjuvant chemoradiotherapy in integrated treatment of cancer of the subperitoneal rectum.

**Results:**

Surgical procedures performed were abdominal perineal resection in 4 cases (3.5%), anterior resection in 89 cases (78%), Hartmann’s procedure in 5 cases (4.4%), and ultralow resection with coloanal anastomosis and diverting stoma in 16 patients (14%).

Local recurrence occurred in 6 patients (5.2%), the overall survival was 71.9% at 5 years and disease-free survival was about 60%.

**Conclusions:**

The effect of pathological downstaging amounted to 58.8%, including cPR. The pathologic complete remission occurred in 8.8% of cases.

The outcomes of neoadjuvant therapy can be achieved when this treatment is associated with correct surgical technique with TME and the prognosis is defined by an anatomopathological examination performed according to Quirke’s protocol.

## 1 Introduction

Over the past two decades, outcomes in the management of rectal cancer have improved thanks to a multidisciplinary approach that involves the surgeon, pathologist, radiotherapist and oncologist. There have been continuous efforts to improve local control and long-term survival in patients with rectal cancer, and the introduction of neoadjuvant therapy protocols in the treatment of this tumor has permitted the downstaging of the neoplasm in many cases.

Neoadjuvant therapy has also led to the increase of number of conservative interventions, as well as improving the prognosis by reducing the incidence of recurrence and increasing survival [1, 2, 3, 4, 5, 6]. On the other hand surgery alone has a high rate of complications (10-65%) and local recurrence (10-29%) [7].

Recently, several studies have generated considerable interest in the neoadjuvant treatment, which consists of the administration of chemotherapy combined with radiation therapy (RT) before surgery, with the aim of enhancing the local action of radiation and to sterilize the nodal micrometastases, thus increasing the possibility of achieving radical oncological surgery and sphincter-saving operations [8, 9, 10, 11, 12, 13].

In patients undergoing pre-operative RT, there is an objective response rate of 65-70% and 5-10% of complete responses; the pre-operative RT significantly reduces the rate of local recurrence and prolongs survival [12,14].

The preoperative chemoradiotherapy has a lower toxicity because it acts at an earlier stage in respect to the development of metastatic cells [15, 16, 17].

In this study, we assessed 114 patients with subperitoneal rectal cancer whounderwent preoperative neoadjuvant chemoradiotherapy and total mesorectal excision (TME), from January 2004 to December 2013 at Department of Advanced Biomedical Sciences, University of Naples Federico II, Naples, Italy. We evaluated through the experience of a multidisciplinary team in an observational real-life retrospective study, and the role of neoadjuvant chemoradiotherapy in the integrated treatment of cancer of the lower rectum in the light of oncology and clinical factors outcomes.

## 2 Methods

From January 2004 to December 2013 at our center were admitted 376 patients (206 male and 170 females) with colorectal cancer in an observational real-life retrospective study. Of these 376, 189 patients had adenocarcinomas of the colon and 187 patients had adenocarcinomas of the rectum. Among them, 150 patients showed a cancer of the rectum localized in the subperitoneal space (<12 cm from the anal verge). The 150 patients with subperitoneal rectal cancer were evaluated (82 male and 68 female, average age 55 years, range 26-75 years) and were submitted to the following examinations: routine blood chemistry, research of tumor markers, colonscopy, endorectal ultrasound, MRI, CT/PET Total body.

Ethical approval: The research related to human use has been complied with all the relevant national regulations, institutional policies and in accordance the tenets of the Helsinki Declaration, and has been approved by the authors' institutional review board or equivalent committee. At the first observation, each patient gave signed informed consent to undergot the surgical procedure and be a part of the clinical research.

The study protocol had the following exclusion criteria: beyond the intraperitoneal tumor site (> 12 cm from the anal verge) (11 cases); histology different from adenocarcinoma (3 squamous cancer of the anus; 2 lymphomas); synchronous primitive multiple cancers (MPM) (6 cases); presence of metastases (8 cases); and T1, T4 and T2N0 (6 cases).

Of the 150 patients, 114 patients were enrolled and treated with neoadjuvant therapy.

The synthesis before and after neoadjuvant treatment of 114 patients is reported in Table 1.

**Table 1:** Demographic, clinical and surgical procedures in 114 patients

**Gender; No (%)**:	
**Male**	65 (57)
**Female**	49 (42.9)

**Range of average age**	55.0 (26-75)

**Follow up (months)**	39.4 (2.2-151.6)

**Tumor level, No (%)**:	
**High**	0
**Mid**	58 (50.9)
**Low**	56 (49.1)

**Surgical Procedure, No (%)**:	
**Abdominal perineal amputation**	4 (3.5)
**Low anterior resection**	89 (78.0)
**Hartmann**	5 (4.4)
**Ultra low anterior resection with**	16 (14.0)
**anastomosis coloanal**	

**The local recurrence at 5 years, No (%)**:	6 (5.2)

**The overall survival at 5 years, No (%)**:	82 (71.9)

**Disease-free survival at 5 years, No (%)**:	68 (59.6)

The treatment given to patients is constituted by one of concomitant chemotherapy scheme [18,19].

### 2.1 External radiation therapy (RT)

Radiation treatment was given using a linear accelerator (6-25 MV) and isocentric technique of 3 fields (one posterior and 2 lateral) with a total dose of 50.4Gy (25 daily fractions of 1.8Gy to the pelvis plus 3 additional daily fractions of 1.8Gy to the tumor). The entire treatment had a duration of 28 days.

### 2.2 Chemotherapy (CHT)

Continuous infusion i.v. with elastomeric pump of 5-fluorouracil at dose of 225 mg/m2 for the duration of the radiation treatment. Two boluses i.v. week of oxaliplatin at dose of 60 mg/m2.

The patients were re-admitted at our center 4-6 weeks after the end of neoadjuvant therapy in order to perform restaging and undergo surgery between 6th and 8th week after the end of preoperative chemoradiotherapy.

For all patients at the time of initial staging, we assessed whether or not to perform a conservative surgery of the sphincter function, essentially based on the distance of the tumor from the anal verge.

The follow-up after surgery was standard with six-monthly checks for at least 5 years.

All patients were followed up by the surgeon, specialist oncologist, and radiotherapist, and were subjected to careful clinical evaluation, blood chemistry with evaluation of the levels of CEA, and chest X-ray.

## 3 Results

We enrolled 114 consecutive patients, 65 were male and 49 female with a mean age 55 years and range 26-75 years. All cases had histologically determined adenocarcinoma of the rectum. They were considered only when the rectal cancer localization was ≤ 12 cm from the anal verge.

All patients underwent clinical-laboratory weekly monitoring during the course of concomitant chemotherapy, which showed no toxic effects, with a performance status of ECOG grade 0 (patient physically active can perform normal activities without restriction). Only two patients presented a moderate neutropenia. All 114 patients completed the set cycle.

The surgical procedures performed were: abdominal perineal resection in 4 cases (3.5%), anterior resection in 89 cases (78%), Hartmann’s procedure in 5 cases (4.4%), and ultralow resection with coloanal anastomosis and diverting stoma in 16 patients (14%) (Table 1).

The mean number of lymph nodes examined was 12; the hospital stay was 9-25 days. Seventeen patients had postoperative complications: postoperative wound dehiscence in 3 (2.6%) patients, anastomotic leak in 9 (7.9%) patients, major cardiovascular complications in 5 (4.4%) patients (Table 2).

**Table 2:** Postoperative complication in 17 patients.

Postoperative complications, n (%):	
Wound dehiscence	3 (2.6%)
Anastomotic leak	9 (7.9%)
Major cardiovascular complications	5 (4.4%)

In all 114 patients the resection margins assessed with pathologic examination of the surgical specimen had no residual tumor cells.

During follow-up, we detected two cases of local recurrence (1.8%) at 6 and 15 months respectively. Both patients had lymph node recurrence after surgery, and one of them developed liver metastases. The local recurrence occurred in 6 patients (5.2%) at 5 years, the overall survival at 5 years was observed in 82 (71.9%) patients and disease-free survival at 5 years in 68 patients (59.6%).

## 4 Conclusion

In recent years, the use of neoadjuvant chemoradiotherapy has increased in the perioperative treatment of rectal cancer [20, 21, 22]. Several studies show that the combination of a “short course” of neoadjuvant radiotherapy and total mesorectal excision results in a better local control than only TME [21]. In addition, the neoadjuvant chemoradiotherapy has been introduced in the treatment of locally advanced rectal cancer to improve the preservation of the anal sphincter [14,22, 23, 24]. In fact, when the rectal cancer is locally advanced at diagnosis, recurrence after surgical resection occurs in a range of 50% to 70% of cases and is characterized by the early onset of distant metastases [25]. The neoadjuvant chemoradiotherapy is part of a safe and efficient multimodal treatment of rectal cancer. It shows significant benefits in terms of reducing the tumor volume and less compromise to the neoplastic lymph node, thus confirming the concept of down staging.

In this study, the effect of pathological down staging amounted to 58.8%, including cPR. The pathologic complete remission occurred in 8.8% of cases. Patients who had a complete pathological remission have had good outcomes in terms of OS and DFS (71,3% and 60 %, respectively).

In locally advanced rectal carcinoma, this combined treatment approach aims to improve resectability, local control and overall survival. It does so by reducing the tumor volume thus favoring surgical resection and longterm oncological results [26, 27, 28,].

In our experience we found a clinical and instrumental down staging in 59 patients (51.75%). This finding is in agreement with literature, the neoadjuvant treatment for carcinoma of the rectum can cause a down staging which permits an increase of conservation of the sphincter function [21, 22, 23, 24,29, 30, 31].

Many trials also show that the neoadjuvant chemoradiotherapy appears associated with a decrease in the rate of local recurrence and to an increase in the overall survival rate [18]. However, these data are not accepted univocally as some authors argue that the neoadjuvant chemoradiotherapy, though it determines a down staging and, sometimes, a complete “eradication” of the tumor histology, is complicated by high toxicity and a high rate of anastomotic leak [32].

In agreement with this finding, in patients subjected to ultralow colo-rectal anastomosis we always made a temporary ileostomy [33].

Some authors emphasize the role of lymph nodes involvement detected on the histological sample. In fact in patients treated with preoperative chemoradiotherapy the presence of positive lymph nodes in the histological sample would be associated with a worse prognosis [34,35]. Hence the importance of intraoperative lymph node metastases [36].

In our study, the preoperative staging of rectal cancer was performed with the digital rectal examination, transrectal ultrasound, colonoscopy and magnetic resonance imaging (MRI).

Nowadays EUS is considered very accurate for T staging, because it can precisely define the degree of tumor penetration into the various layers of the rectal wall [37, 38, 39, 40, 41]. MRI is the best method to define distance of the mesorectal fascia from the lateral margin of the tumor and so to determine which patients can benefit from neoadjuvant therapy. Moreover, its accuracy is greater than ultra-sonography in identifying the tumors [35, 42, 43, 44, 45, 46, 47].

The pathological complete response occurred in 10 patients (8.8%), like observed by Grann et al. in 9% of cases [9]. However, if the cancer has been shown to have a pathological downstaging, the latter can provide related benefits to the resection of the tumor and the improvement of the oncological results [26, 27].

The final objective of preoperative chemoradiotherapy was the pathological complete remission. The best outcomes of neoadjuvant therapy can be achieved when this treatment is associated with a correct surgical technique with TME and the prognosis is defined by an anatomopathological examination performed according to Quirke’s protocol.
